# Prevalence and risk factors for non-use of antenatal care visits: analysis of the 2010 South Sudan household survey

**DOI:** 10.1186/s12884-015-0491-6

**Published:** 2015-03-26

**Authors:** Ngatho S Mugo, Michael J Dibley, Kingsley E Agho

**Affiliations:** 1 School of Public Health, Edward Ford Building (A27), University of Sydney, Sydney, NSW 2006 Australia; 2 School of Science and Health, Building (24), University of Western Sydney, Locked Bag 1797, Penrith, NSW 2751 Australia

**Keywords:** Antenatal care, Pregnancy complications, Socioeconomic factors, Mortality, South Sudan

## Abstract

**Background:**

Antenatal care (ANC) is a preventive public health intervention to ensure healthy pregnancy outcomes and improve survival and health of newborns. In South Sudan, about 40% of pregnant women use ANC, however, the frequency of the ANC checks falls short of the recommended four visits. Hence, this study examined potential risk factors associated with non-use of ANC in South Sudan.

**Method:**

Data for this analysis was from the 2010 South Sudan Household Survey second round, which was a nationally representative stratified cluster sample survey. The study included information from 3504 women aged 15–49 years who had given birth within two years preceding the survey. Non-use of ANC was examined against sixteen potential risk factors, using simple and multiple logistic regression analyses adjusted for cluster sampling survey design.

**Results:**

The prevalence of non-use of ANC was 58% [95% confidence interval (CI): (55.7, 59.8)], the prevalence of 1–3 ANC visits was 24% [95% CI: (22.7, 26.7)] and that for 4 or more visits was 18% [95% CI: (16.3, 19.3)]. After adjusting for potential confounding factors, geographic regions, polygamy status [adjusted odds ratio (AOR) = 1.23; 95% CI: (1.00, 1.51), p = 0.047 for a husband with more than one wife], mother’s literacy [AOR = 1.79; 95% CI: (1.31, 2.45), p = 0.001 for illiterate mothers], and knowledge on a newborns’ danger signs [AOR = 1.77; 95% CI (1.03, 3.05), p = 0.040 for mothers who had limited knowledge of a newborns’ danger signs] were significantly associated with non-use of ANC.

**Conclusions:**

Overall improvement of women’s access to the recommended number of ANC visits is needed in South Sudan. Strategies to encourage Southern Sudanese women to pursue education as well as to raise awareness about the importance of ANC services are essential. It is also important to prioritize strategies to increase access to health care services in rural areas as well as developing strategies to reduce the financial burden associated with maternal health services.

## Background

Underutilization of ANC services among pregnant women in many low and middle-income counties has been a major public health issues with only 51% attending four or more ANC visits [1]. However, the rate of progress has been slowest in sub-Saharan Africa with about 44% women receiving at least four or more ANC visits [1]. ANC from a medically trained provider is an essential service for pregnant women aimed at ensuring healthy pregnancy outcomes and improving survival rates of the newborn [2]. Effective ANC links the pregnant woman and her family to formal health systems, enhances the chance of using skilled birth attendants at delivery and contributes to good health throughout pregnancy [3]. Timely access to ANC is one of the most effective methods to improve pregnancy outcomes in less-developed countries [4,5].

Pregnant woman in South Sudan have poor health status with significant urban–rural and regional disparities [6-8]. The Ministry of Health of South Sudan has adopted a minimum of four ANC visits as recommended by the WHO to improve the wellbeing of women and their infants [4,9]. However, the proportion of women receiving at least one ANC examination from any skilled health provider was only 26% in 2006, leaving about 74% of pregnant women without any ANC [10]. WHO recently reported the maternal mortality ratio in South Sudan as 2,054 per 100,000 [11]. These high levels of maternal mortality in Southern Sudan are associated with poor access to quality reproductive health services, including ANC services, trained health personnel at delivery and family planning services [9,12].

In South Sudan the prolonged conflict, which has lasted over two decades, has destroyed much of the health services with currently over 40% of health facilities not operative [13,14]. As a result, pregnant women in South Sudan have limited access to, and availability of maternal health services [9].

Several studies have examined risk factors affecting utilization of ANC services [15-19]. In South Sudan the factors affecting ANC utilization, the social dynamics, barriers and use of maternal health care services are not adequately understood. Therefore, this analysis examined the prevalence of non-use of ANC services, and the associated risk factors. The results from this study will help public health and policy makers to develop interventions aimed at improving access to maternal and neonatal health services to reduce maternal and child mortality in South Sudan.

## Methods

### Data sources

The data set used in this research was collected during the 2010 South Sudan Household Health Survey second round (SSHHSII). The SSHHSII is a nationally representative, stratified, cluster sample survey, covering the entire population of South Sudan. It aimed to collect health and related indicators essential for identifying the health needs of women and children and for establishing priorities for evidence-based planning, decision-making and reporting. The survey was largely based on the UNICEF’s Multiple Indicator Cluster Survey (MICS) methodology [9]. The MICS is a five to three -year periodic survey programme developed by UNICEF to assist countries to fill data gaps in monitoring the situation of women and children. The survey comprises of four questionnaires: household, women and men aged 15–49 years and children younger than 5 years. The women’s questionnaire includes questions about the women’s demographic characteristics, reproductive history, pregnancy, antenatal care, as well as immunization. Details of the SHHSII sampling methods have been reported elsewhere [9].

### Sample size

The sample size for the SSHHSII was calculated as 10,000 households using the prevalence of under-five child diarrhea as the key indicator assuming a prevalence of 20%, a design effect of 1.5, 16% of the total population to be under-five children, and a participation rate of 90%. The results reported in this paper were based on data from 3,504 women with the primary outcome non-use of antenatal care. We estimate that this sample has 80% power to detect an odds ratio of at least 1.24, or a difference of prevalence of 5.7%, assuming an alpha level of 5%, prevalence of non-use of ANC of 60%, a design effect of 1.25 (based on other surveys) [20], and a total sample of 2800, which was obtained by dividing 3500 by the value for the design effect. We consider this sufficient statistical power to examine differences in non-use of ANC that would be of public health significance.

### Study population

**Figure 1:**
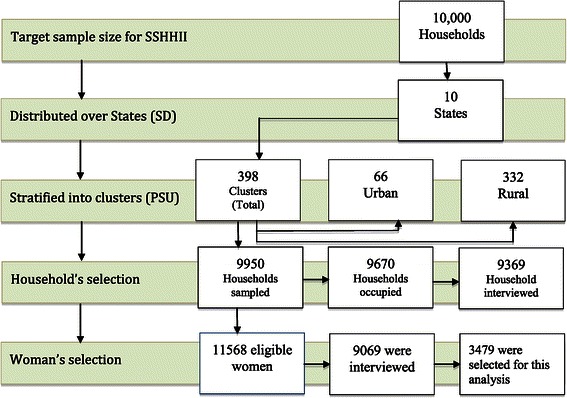
**A multiple stage methodology process for selection of woman at the household level, 2010-SSHHSII.** State: is the sampling domain. Cluster: is the primary sampling unit (PSU).

The study population for this analysis was limited to women aged 15–49 who gave birth in the last two years preceding the survey. Figure 1 shows the selection process of the women at the household level. Among 11,568 of eligible woman there were 9,069 who were interviewed with a response rate of 78.4%. Information on the ANC visits was collected for the last birth from women, who had more than one birth in the last two years preceding the survey. A total of 3,504 women had at least one birth in the two years preceding the survey.

### Variables

Non-use of ANC services was the primary outcome variable used in the analysis of the SSHHSII. It was categorized into three groups consisting of: 1) those women who had ANC checks by non-skilled providers, and those who had no ANC, 2) those who had between 1 to 3 ANC checks by skilled providers, and 3) those who attended 4 or more ANC checks by skilled providers. The World Health Organization defines ANC as “care before birth”, and includes education, counseling, screening and treatment to monitor and to promote the well being of both mother and baby [21]. For the purpose of this analysis, ANC service refers to any pregnancy-related services provided by skilled health personnel, such as doctors, nurse-midwives, midwives and health visitors [9] whereas non-skilled ANC services refers to women receiving no ANC services at all or woman receiving any pregnancy-related services provided by non-SBAs, such as, traditional birth attendants, community health workers, relatives or friends.

**Figure 2:**
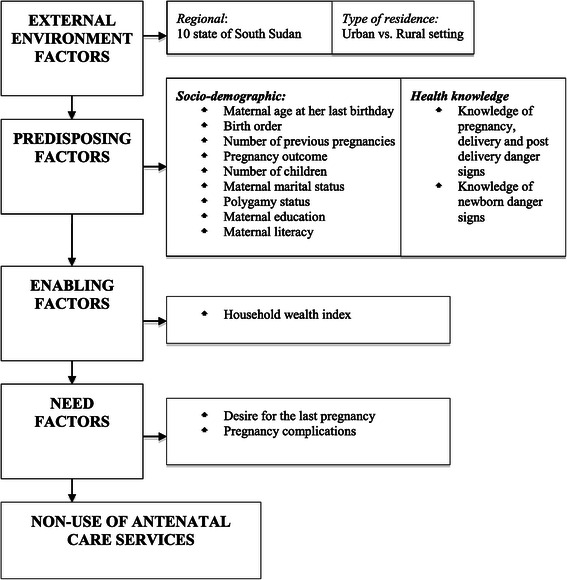
**Theoretical framework of a range of risk factors associated with non-use of antenatal care in South Sudan.** This framework adopted from Anderson behavioural model.

We modified the Andersen behavioural model framework for health services utilization to understand the factors that determine pregnant women’s use of health care services [22]. Figure 2 shows the modified Andersen behavioural model conceptual framework and all the variables included in this analysis. The possible risk factors associated with non-use of ANC services were categorized into four main groups; namely (1) external environment including health services characteristics of the regions and living in rural/ urban, (2) predisposing factors such as maternal characteristics that existed before the onset of the need for ANC services, (3) enabling factors that facilitate the pregnant women to receive ANC services, and (4) need factors that indicate the potential for adverse ANC outcomes. Using the Andersen model, sixteen potential risk factors associated with non-use of ANC services were identified and categorized into external environment, predisposing, enabling and need factors.

### Statistical analysis

Descriptive analyses were performed using the STATA/MP version 12 (StataCorp, College Station, TX, USA); ‘Svy’ commands were used to allow for adjustments for the cluster sampling design, sampling weights and the calculation of standard errors. The Taylor series linearization method was used in the analysis when calculating confidence intervals around prevalence estimates. Cross tabulations were generated to describe the frequencies and confidence intervals of ANC services across independent variables, and the statistical significance was tested using chi-squared tests.

To determine the risk factors for non-use of ANC services, the outcome variable was expressed in a binary form, that is, category 0 for 1 to 4+ ANC visits and category 1 for no ANC visits. In the univariate and bivariate logistic regression, which was adjusted for the effects of the sampling design and weighted, the odds ratios with 95% confidence intervals were calculated to determine the unadjusted risk of independent variables on non-use of ANC services. Multiple logistic regression was used in a backwards elimination model in order to identify the factors significantly associated with the study outcome. At the start, all variables were included in the model and backward elimination process was employed to remove non-significant variables. Only variables with statistical significance of p < 0.05 were retained in the final model. In the final model, we tested and reported any co-linearity, and calculated the odds ratios with 95% confidence intervals in order to assess the adjusted risk of the independent variables.

### Ethical approval

The ethics committee of the Ministry of Health, Government of South Sudan, reviewed and approved this research study. All respondents to the survey provided verbal informed consent; consent for children was obtained through the parents, caregivers or guardians. The dataset of SSHHII is not available as a public domain survey dataset. The first author requested the access to the data from Director of Health Social and Demographic Statistics and from the Ministry of Health of South Sudan, and access was granted to use the data for research.

## Results

**Figure 3:**
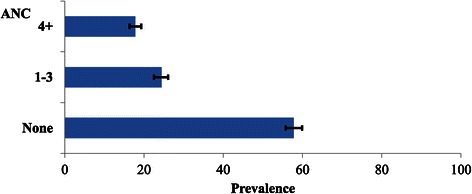
**The prevalence of maternal use of antenatal care by number of visits, in South Sudan.**

Our study population consisted of 3,504 (weighted total) women aged between 15–49 years who had given birth in the two years preceding the survey distributed across the 10 regions of South Sudan. The prevalence of women who did not use any form of ANC services was found to be 58% [95% CI: (55.7, 59.8)]. The prevalence of women who made less than four ANC visits was 24% [95% CI: (22.7, 26.7)] and that of women who made the recommended number of ANC visits was about 18% [95% CI: (16.3, 19.3)] (see Figure 3).

### Characteristics of the study sample

Table 1 describes the baseline characteristics of the risk factors. More than three-quarters (77%) of the women interviewed resided in rural areas and the majority of them (72%) were aged between 20–34 years. About 87% of them had a first birth order child. Nearly all of the women (90%) were illiterate and 78% had no formal education. Few women showed good knowledge on newborn danger signs (4.0%) and knowledge on the obstetric danger signs and symptoms associated with pregnancy, delivery and post-delivery complications (4.2%). Slightly more than one third of the women experienced at least one complication during the course of pregnancy. Only 18% of the women attend the recommended 4 or more ANC visits during pregnancy. About 57% of the women received ANC services from unskilled health personnel. Almost 58% of the women had not attended any form of ANC during the course of their pregnancy. More than four-fifths (85%) of the woman wanted their last pregnancy and about 77% were currently married.

**Table 1:** **Baseline characteristics of factors associated with maternal utilization of ANC health services, categorized by the external environment, predisposing, enabling and need factors in South Sudan, SSHHSII 2010 (n = 3504)**

VARIABLES	n	%
**1. EXTERNAL ENVIRONMENTAL FACTORS**		
***Geographical Region (state)***		
Upper Nile	464	13.3
Jounglei	452	12.9
Unity	205	5.8
Warap	480	13.7
Northern Bahr el Ghazal	280	8.0
Western Bahr el Ghazal	138	3.9
Lakes	280	8.0
Western Equatoria	313	8.9
Central Equatoria	500	14.3
Eastern Equatoria	392	11.2
***Type of residence (total)***		
Urban	808	23.0
Rural	2697	77.0
**2. PREDISPOSING FACTORS**		
**Socio-demographic characteristic**		
***Maternal age at her last birthday (years)***		
15-19	272	7.8
20-34	2520	71.9
35-49	713	20.3
***Birth order***		
1st birth	2794	86.5
2nd birth	368	11.4
3rd + birth	68	2.1
***Number of previous pregnancies***		
1 pregnancy	3113	95.1
2+ pregnancy	161	4.9
**Pregnancy outcome**		
Live birth	3062	96.7
Other (Still birth, Miscarriage, Currently pregnant)	105	3.3
***Number of children***		
1-2 children	1211	34.6
3-4 children	1157	33.0
5 children and more	1136	32.4
***Maternal marital status***		
Currently married	2702	77.1
Formerly married	582	16.6
Never married (Single)	220	6.3
**Polygamy status**		
Husband have one wife	1889	59.0
Husband have more than one wife	1313	41.0
***Maternal education***		
No education	2745	78.4
Primary education	615	17.6
Secondary + education	143	4.1
***Maternal literacy***		
Able to read	334	10.0
Unable to read	3018	90.0
**Health knowledge**		
***Knowledge of obstetric danger signs during pregnancy, delivery and post delivery***		
Good for (correct answer 8 or more)	147	4.2
Fair for (correct answer between 5–7)	170	4.8
Bad for (correct answer less than 5)	3188	91.0
***Knowledge on newborn danger signs***		
Good for (correct answer 8 or more)	140	4.0
Fair for (correct answer between 5–7)	180	5.1
Bad for (correct answer less than 5)	3184	90.9
**3. ENABLING FACTORS**		
***Household wealth index***		
Poorest	671	19.2
Poorer	704	20.1
Middle	654	18.7
Richer	747	21.3
Richest	728	20.8
**4. NEED FACTORS**		
***Desire for last pregnancy***		
Wanted to get pregnant then	2995	85.5
Wanted to get pregnant later	314	9.0
Never wanted to get pregnant	106	3.0
***Pregnancy complications***		
Yes with 1–2 complications	1240	35.4
Yes with 3 and more complications	1140	32.5
No without complications	1124	32.1

### Factors associated with non-use of the recommended number of ANC visits

Table 2 shows the external environment, predisposing, enabling and need factors were significantly associated with use of ANC visits in South Sudan. The analysis shows that the residence of pregnant women in Warab, Junglei and Unity regions was strongly associated with maternal non-use of ANC visits compared to their counterparts from the remaining regions. The table also shows that pregnant women from rural areas were more likely to underutilize ANC services compared to their counterparts from urban areas. The non-use of ANC services was also significantly higher among illiterate women, women who had no formal education, those whose husbands had more than one wife, and among never married (single) women. Among the other socio-demographic factors, maternal age was negatively associated with the outcome variable. For instance, older women (aged 35–49 years) tend not to utilise ANC visits during the course of their pregnancy compared to younger women (aged 15–19 years). The pregnancy outcomes of having stillbirths or miscarriages were significantly higher among mothers who did not utilise ANC services. Knowledge of obstetric danger signs during pregnancy, delivery and post-delivery, as well as knowledge on newborn danger signs were other significant factors that were strongly associated with non-use of ANC services. Enabling resources, such as the household wealth index, were significantly associated with non-use of ANC services. For instance women from the top two household wealth quintiles had an increased utilization of the recommended number of four or more ANC visits compared to their counterparts from the lowest two quintiles. Women who never experienced pregnancy complications were strongly associated with non-use of ANC services compared to their counterparts who experienced more than one complication. Utilization of unskilled health personnel was highly significant among non-users of ANC with a prevalence of 94%.

**Table 2:** **Number of Antenatal care visits according to external environment, predisposing, enabling and need factors in South Sudan, SSHHSII 2010 (n = 3504)**

VARIABLE	Number of antenatal care visits						
	None		1-3 visits		4+ visits		P
	%	[95% CI]	%	[95% CI]	%	[95% CI]	
**1. EXTERNAL ENVIRONMENTAL FACTORS**							
***Geographical Region (state)***							
Upper Nile	57.7	(52.1, 63.1)	22.3	(18.7, 26.3)	20.1	(15.9, 25.1)	
Jounglei	72.5	(66.0, 78.2)	18.7	(14.2, 24.2)	8.8	(5.6, 13.6)	
Unity	68.3	(61.0, 74.8)	18.9	(14.1, 24.8)	12.8	(8.9, 18.1)	
Warap	78.8	(73.8, 83.1)	15.7	(11.9, 20.6)	5.5	(3.2, 9.1)	
Northern Bahr el Ghazal	58.2	(51.0, 65.0)	29.5	(22.7, 37.3)	12.3	(9.0, 16.7)	
Western Bahr el Ghazal	51.3	(46.2, 56.5)	20.9	(16.7, 25.9)	27.7	(23.8, 32.1)	
Lakes	53.7	(47.8, 59.5)	30.3	(25.9, 35.1)	16.0	(11.5, 21.7)	
Western Equatoria	41.9	(33.6, 50.7)	31.2	(23.1, 40.5)	26.9	(19.6, 35.8)	
Central Equatoria	33.5	(28.6, 38.8)	33.9	(28.3, 40.0)	32.6	(27.7, 37.9)	
Eastern Equatoria	58.5	(52.1, 64.6)	23.2	(18.6, 28.4)	18.4	(13.2, 25.1)	<0.001
***Type of resident (total)***							
Urban	40.8	(36.5, 45.2)	28.4	(23.1, 34.3)	30.9	(26.3, 35.9)	
Rural	62.9	(60.6, 65.2)	23.3	(21.7,25.0)	13.8	(12.6, 15.2)	<0.001
**2. PREDIPOSING FACTORS**							
**Scio-Demographic characteristic**							
***Maternal age at her last birthday (years)***							
15-19 years	52.3	(44.2, 60.3)	22.6	(17.7, 28.3)	25.1	(19.3, 32.1)	
20-34 years	57.0	(54.4, 59.6)	25.3	(23.2, 27.5)	17.7	(15.9, 19.7)	
35-49 years	62.7	(58.8, 66.5)	22.1	(18.6, 26.2)	15.1	(12.3, 18.5)	0.0156
***Birth order***							
1st birth	59.4	(57.2, 61.6)	24.0	(22.1, 25.9)	15.6	(15.3, 18.0)	
2nd birth	47.8	(41.6, 54.2)	27.9	(22.2, 34.5)	24.2	(18.8, 30.6)	
3rd + birth	26.0	(15.4, 40.5)	34.0	(20.9, 50.0)	40.0	(25.7, 56.3)	<0.001
***Number of previous pregnancies***							
1 pregnancy	55.3	(53.2, 57.3)	26.0	(24.1, 27.9)	18.8	(17.3, 20.3)	
2+ pregnancy	46.7	(38.3, 55.3)	29.8	(23.7, 36.7)	23.5	(17.0, 31.6)	<0.001
***Pregnancy outcome***							
Live birth	54.9	(52.9, 56.9)	26.1	(24.2, 28.1)	19.0	(17.6, 20.6)	
Other (Still birth, Miscarriage, Currently pregnant)	62.8	(51.9, 72.4)	17.8	(11.0, 27.6]	19.4	(11.6, 30.6)	<0.001
***Number of children***							
1-2 children	55.3	(51.6, 56.0)	25.3	(22.3, 28.6)	19.4	(16.7, 22.4)	
3-4 children	61.6	(58.2, 64.9)	22.8	(19.9, 25.9)	15.6	(13.3, 18.4)	
5 children and more	56.6	(53.5, 59.7)	25.2	(22.2, 28.5)	18.2	(15.8, 20.9)	0.1245
***Maternal marital status***							
Currently married	55.6	(53.2, 58.0)	25.3	(23.3, 27.4)	19.1	(17.4, 21.0)	
Formerly married	66.0	(60.9, 70.8)	21.2	(17.2, 25.8)	12.8	(10.0, 16.3)	
Never married (Single)	63.4	(55.6, 70.5)	22.5	(16.2, 30.5)	14.1	(9.4, 20.6)	0.0022
***Polygamy status***							
Husband have one wife	54.5	(52.0, 57.0)	25.3	(22.9, 27.9)	20.2	(17.9, 22,7)	
Husband have more than one wife	61.2	(58.1, 64.0)	23.8	(21.4, 26.5)	15.0	(13.0, 17.3)	0.0033
***Maternal education***							
No education	64.8	(62.6, 67.0)	22.4	(20.7, 24.2)	12.8	(11.5, 14.3)	
Primary education	33.0	(28.8, 37.5)	33.2	(28.6, 38.2)	33.8	(29.1, 38.8)	
Secondary + education	29.8	(19.8, 42.1)	26.1	(18.6, 35.2)	44.2	(34.4, 54.5)	<0.001
***Maternal literacy***							
Able to read	36.5	(30.8, 42.6)	34.1	(27.7, 41.2)	29.4	(23.9, 35.6)	
Unable to read	61.4	(59.2, 63.6)	23.3	(21.5, 25.2)	15.3	(13.9, 16.7)	<0.001
**Health knowledge**							
***Knowledge of obstetric danger signs during pregnancy, delivery and post delivery***							
Good for (correct answer 8 or more)	42.6	(33.9, 51.7)	30.7	(23.1, 39.4)	26.8	(19.2, 36.0)	
Fair for (correct answer between 5–7)	52.1	(42.9, 61.2)	28.6	(21.0, 37.7)	19.3	(11.6, 30.4)	
Bad for (correct answer less than 5)	58.8	(56.6, 61.0)	23.9	(22.0, 25.9)	17.3	(15.9, 18.7)	0.0247
***Knowledge on newborn danger signs***							
Good for (correct answer 8 or more)	45.3	(33.8, 57.3)	25.6	(17.0, 36.7)	29.1	(19.6, 40.9)	
Fair for (correct answer between 5–7)	37.8	(29.9, 46.3)	42.1	(32.7, 52.0)	20.2	(13.8, 28.6)	
Bad for (correct answer less than 5)	59.5	(57.3, 61.6)	23.4	(21.6, 25.2)	17.1	(15.6, 18.7)	<0.001
**3. ENABLING FACTORS**							
***Household wealth index***							
Poorest	74.5	(70.3, 78.3)	20.3	(17.3, 23.7)	5.2	(3.3, 8.2)	
Poorer	66.8	(62.8, 70.6)	20.7	(17.6, 24.2)	12.5	(10.3, 15.0)	
Middle	62.6	(59.3, 65.9)	23.9	(20.7, 27.5)	13.4	(10.8, 16.7)	
Richer	52.5	(48.0, 56.9)	26.5	(23.2, 30.2)	21.0	(17.9, 24.6)	
Richest	34.8	(29.6, 40.4)	30.2	(25.0, 36.0)	35.0	(30.3, 40.0)	<0.001
**4. NEED FACTORS**							
***Desire for last pregnancy***							
Wanted to get pregnant then	59.1	(57.1, 61.2)	23.7	(21.9, 25.7)	17.1	(15.6, 18.7)	
Wanted to get pregnant later	47.9	(40.0, 55.8)	29.0	(22.9, 35.8)	23.2	(17.8, 29.6)	
Never wanted to get pregnant	42.9	(33.6, 52.8)	34.5	(25.7, 44.5)	22.6	(15.3, 32.0)	0.007
***Pregnancy complications***							
Yes with 1–2 complications	49.5	(46.6, 52.5)	28.6	(25.6, 31.8)	21.9	(19.5, 24.4)	
Yes with 3 and more complications	52.6	(49.7, 55.4)	27.1	(24.3, 30.0)	20.4	(18.1, 22.9)	
No without complications	72.3	(68.8, 75.5)	17.2	(14.9, 19.7)	10.6	(8.7, 12.7)	<0.001

Table 3 presents the adjusted and unadjusted odds ratios for the factors associated with maternal non-use of ANC visits. The analysis shows that socio-demographic factors including maternal level of education, maternal age, number of children and their birth order, number of previous pregnancies and the outcomes, and marital status were associated with the use of ANC services in South Sudan. Geographical region was significantly associated with women’s non-use of ANC services. For instance, women who resided in Jounglei [AOR = 1.76; 95% CI: (1.19, 2.60), P = 0.005], Warab [AOR = 1.66; 95% CI: (1.16, 2.23), P = 0.127] and Unity [AOR = 1.42; 95% CI: (0.90, 2.23), P = 0.127] regions were more likely not to utilize ANC services compared to other regions of South Sudan. Among the socio-demographic factors, the odds of non-use of ANC services increased significantly for women whose husbands had more than one wife and among illiterate women (woman who were unable to read). Knowledge on newborn danger signs was another significant variable associated with non-use of ANC.

**Table 3:** **Unadjusted and Adjusted odds ratios for factors associated with maternal non-use of ANC services, in South Sudan, SSHHSII 2010 (n = 3504)**

VARIABLE	Unadjusted odds ratio			Adjusted odds ratio (AOR)*#		
	OR	[95% CI]	P value	AOR	[95% CI]	P value
**1. EXTERNAL ENVIRONMENTAL FACTORS**						
***Geographical Region (state)***						
Upper Nile	1.00			1.00		
Jounglei	1.94	(1.37, 2.74)	<0.001	1.76	(1.19, 2.60)	0.005
Unity	1.58	(1.01, 2.47)	0.044	1.42	(0.90, 2.23)	0.127
Warap	2.73	(1.95, 3.82)	<0.001	1.66	(1.16, 2.38)	0.006
Northern Bahr el Ghazal	1.02	(0.69, 1.52)	0.913	0.81	(0.52, 1.27)	0.354
Western Bahr el Ghazal	0.77	(0.60, 1.01)	0.055	0.73	(0.54, 0.98)	0.037
Lakes	0.85	(0.60, 1.20)	0.349	0.63	(0.41, 0.97)	0.036
Western Equatoria	0.53	(0.34, 0.83)	0.006	0.43	(0.26, 0.69)	0.001
Central Equatoria	0.37	(0.27, 0.83)	<0.001	0.42	(0.29, 0.62)	<0.001
Eastern Equatoria	1.03	(0.74, 1.45)	0.849	0.93	(0.66,1.32)	0.698
***Type of resident (total)***						
Urban	1.00					
Rural	2.46	(2.01, 3.03)	<0.001			
**2. PREDISPOSING FACTORS**						
**Socio-demographic characteristic**						
***Maternal age at her last birthday (years)***						
15-19 years	1.00					
20-34 years	1.21	(0.85, 1.71)	0.278			
35-49 years	1.53	(1.08, 2.17)	0.017			
***Birth order***						
1st birth	1.00					
2nd birth	0.67	(0.52, 0.86)	0.003			
3rd + birth	0.52	(0.26, 1.04)	0.063			
***Number of previous pregnancies***						
1 pregnancy	1.00					
2+ pregnancy	0.71	(0.49, 1.02)	0.064			
***Pregnancy outcome***						
Live birth	1.00					
Other (Still birth, Miscarriage, Currently pregnant)	1.38	(0.86, 2.22)	0.172			
***Number of children***						
1-2 children	1.00					
3-4 children	1.29	(1.04, 1.61)	0.021			
5 children and more	1.05	(0.84, 1.32)	0.642			
***Maternal marital status***						
Currently married	1.00					
Formerly married	1.55	(1.20, 2.01)	0.001			
Never married (Single)	1.38	(1.02, 1.88)	0.040			
***Polygamy status***						
Husband have one wife	1.00			1.00		
Husband have more than one wife	1.31	(1.12, 1.54)	0.001	1.23	(1.00, 1.51)	0.047
***Maternal education***						
No education	1.00					
Primary education	0.27	(0.22, 0.33)	<0.001			
Secondary + education	0.23	(0.13, 0.39)	<0.001			
***Maternal literacy***						
Able to read	1.00			1.00		
Unable to read	2.77	(2.09, 3.67)	<0.001	1.79	(1.31, 2.45)	0.001
**Health knowledge**						
***Knowledge of obstetric danger signs during pregnancy, delivery and post delivery***						
Good for (correct answer 8 or more)	1.00					
Fair for (correct answer between 5–7)	1.47	(0.89, 2.43)	0.132			
Bad for (correct answer less than 5)	1.93	(1.34, 2.78)	0.001			
***Knowledge on newborn danger signs***						
Good for (correct answer 8 or more)	1.00			1.00		
Fair for (correct answer between 5–7)	0.73	(0.41, 1.31)	0.288	0.81	(0.40, 1.63)	0.545
Bad for (correct answer less than 5)	1.77	(1.11, 2.84)	0.018	1.77	(1.03, 3.05)	0.040
**3. ENABLING FACTORS**						
***Household wealth index***						
Poorest	1.00					
Poorer	0.69	(0.51, 0.93)	0.015			
Middle	0.57	(0.44, 0.74)	<0.001			
Richer	0.38	(0.29, 0.50)	<0.001			
Richest	0.18	(0.13, 0.25)	<0.001			
**4. NEED FACTORS**						
***Desire for last pregnancy***						
Wanted to get pregnant then	1.00					
Wanted to get pregnant later	0.63	(0.46, 0.88)	0.008			
Never wanted to get pregnant	0.52	(0.36, 0.75)	0.001			
***Pregnancy complications***						
Yes with 1–2 complications	1.00					
Yes with 3 and more complications	1.13	(0.95, 1.34)	0.16			
No without complications	2.65	(2.20, 3.19)	<0.001			

*632 (18%) missing information was not included in the multivariate analysis.

# Independent variables adjusted for are: geographical region (state); type of residence (total); maternal age at her last birthday (years); birth order; number of previous pregnancy; pregnancy outcome; number of children; maternal marital status; polygamy status; maternal education; maternal literacy; knowledge of obstetric danger signs during pregnancy, delivery and post delivery; knowledge on newborn danger signs; household wealth index; desire for last pregnancy; pregnancy complications.

## Discussion

In South Sudan, the main factors that pose risks to non-use of ANC services were: geographical region, the husband’s polygamy status, woman’s literacy and place of residence. The odds of non-use of ANC services were higher in some regions of South Sudan such as Jounglei, Warab and Unity. The risk of not using ANC services was higher among women with no formal education and those from poor households and among older women. The risk of non-use of ANC services was also higher among women who desired to become pregnant, and those who did not experience any pregnancy complications.

This paper is one of the first articles to describe the risk factors for non-use of ANC services in South Sudan. The paper reports potential risk factors that were correlated with non-use of ANC services. The sampling method, appropriate adjustment for sampling design in the analysis including sampling weights, and an adequate response rate (78%) to the survey interviews are important strengths of this survey. This analysis was based on a nationally representative survey SSHHSII 2010, covering the total population of South Sudan. To minimize the potential recall bias, data on the most recent births were obtained from the women who had given birth only during the two years preceding the survey. Also, due to the large size of the survey, we were able to examine a variety of risk factors associated with non-use of ANC services among women in South Sudan, across the external environment, predisposing, enabling and need factors. There were some limitations to the analysis of the data and these should be noted when interpreting the results. The cross-sectional study design restricted the interpretation of the causality of the risk factors associated with non-use of ANC services. Even though the data were collected within 2 years of the preceding survey, it is still subject to recall biases. The recall bias may have occurred because the information collected relied on the woman’s recall ability about her pregnancy. The potential risk factor variables included in this analysis were based on the availability of the information that was found in SSHHSII. As a result, this analysis did not cover many possible risk factors from the Andersen behavioural model framework such as the availability and accessibility of ANC services, content of ANC services and the users’ satisfaction with services. However, we believe these limitations should not have a significant impact on the validity of the study.

Recent studies in other countries have found regional differentials in the under-utilization of ANC services [23,24]. We found that women who resided in Jounglei, Warab and Unity remained highly disadvantaged with increased odds of under-utilising ANC services compared to their counterparts in other regions of South Sudan. The non-use of ANC services could be attributed to the lack of these services or lack of easy access to them in these geographic locations. For instance in Jonglei the long history of inter-ethnic violence among the cattle raiding groups from Lou Nuer, Murle, and Dinka has contributed to overall poor health development, infrastructure and a lack of security [25]. These groups are often illiterate, poor and with low socio economic status. In Unity, Jonglei and Warab regions, most mothers, seeking health services spend two days travelling to health centers [26]. Hence the Government of South Sudan and stakeholders needs to implement mobile clinical services in these remote and rural areas in order to improve ANC services.

One of the key findings of our study was the high risk of non-use of ANC services among woman whose husbands had more than one wife. This finding is similar to that reported in a study conducted in Uganda [27]. Women need support from their husbands to utilize ANC. For women in a polygamous relationship, her husband’s attention is divided between his wives, and therefore he would have less time to pay attention to the needs of each of his wives. In order to understand the barriers on the use of ANC services among women living in polygamous marriages, qualitative research study is needed to understand such barriers. Furthermore, husbands should be educated on the importance of ANC services during pregnancy, and should support and encourage their wives to utilize such services. The Government of South Sudan should also promote educational campaigns targeting both men and women of reproductive age about the importance of ANC services.

Our study found that illiterate mothers had the worst maternal health outcomes compared to their literate counterparts in terms of access and utilization of recommended number of ANC visits. It is also unlikely that illiterate women would seek out quality ANC services; they also lack the essential knowledge that might help them use health care inputs that offer better maternal health care services [28].

The women’s level of education was found to be a risk factor for the non-utilization of ANC services in our study with greater use of services as levels of education increased among women. Previous studies have also found maternal education essential not only for ANC attendance in general, but also in influencing the utilization of antenatal care content [27,29-32]. Several other studies have found a positive and significant association between maternal education and utilization of maternal health services [33-36]. Lack of education has hindered South Sudan women from receiving, seeking and communicating information concerning their health that could help prevent maternal and newborn deaths [37]. These findings show that maternal education can be instrumental in enabling women to gain the knowledge, confidence, skills, and capability to make decisions about their own health and the health of their unborn babies. Therefore the government of South Sudan and other stakeholders should focus on enhancing female education beyond secondary level in order to attain favourable maternal health outcomes in the future. Interventions aimed at mitigating the conditions that lead girls to drop out of school early should be intensified.

We also performed additional analysis to investigate the association between maternal education and literacy. Of those women who were illiterate, 82% of them had no education. Also our analysis indicated that most of the illiterate women (70%) lived in rural areas. Similar findings in developing countries indicate that women who underutilize maternity care are often poor, illiterate, and unmarried, with limited knowledge of maternity care services [38].

In this study, we found that women living in rural areas were less likely to use ANC services, compared to their counterparts in urban areas. This finding is similar to studies conducted by other investigators [39-41], which have reported that non-use of antenatal services was significantly higher among rural women compared to their urban counterparts. Other factors such as higher quality of care, shorter walk-time to health facilities and the woman’s education were significant determinants of routine use of ANC [41]. A study from Ghana [17] found that about a third of the rural population travel long distances (more than 5 km) to reach ANC services. Thus, distance to maternal health services and transportation problems may greatly reduce access to ANC services in rural areas of South Sudan.

The findings from this study could be attributed to the lack of most of the services items of ANC in the rural areas. This may also be brought about by a low demand for ANC in the rural areas. The low demand for the ANC services by these rural women may be due to poverty; and the low demand would explain why health centres would not stock the appropriate items for ANC. Since the majority (80%) of the population of South Sudan reside in rural areas [12], the government and other stakeholders should make efforts to improve facilities and infrastructure (such as better access to paved roads as well as adequate number of maternal health services) in these deprived areas [42].

Our findings also underscore the significance of socio-economic level in influencing the use of ANC services in South Sudan. We found that women in the highest household wealth quintile were more likely to use ANC services, compared to those in the poorest. Other investigators have also underlined the importance of household wealth status in influencing maternal health-seeking behaviour [31,32,40,43]. In our final model, when we replaced literacy with household wealth, it was significant, which suggested there was co-linearity between literacy and household wealth. We believe that household wealth is highly associated with maternal non-use of ANC services in South Sudan since the use of services is associated with financial costs of transportation, physician and facility fees, and the cost of medications. The financial cost of receiving care is a major constraint in South Sudan as over 50% of the population lives below the poverty line, with the majority living in rural settings [12]. In our analysis, women from the wealthy households were those who were literate, had secondary education or higher education, and resided in urban areas. Several other studies have shown that household wealth is positively associated with maternal utilization of health services for delivery [39,44]. The South Sudan government should implement policies that would ensure that all women, irrespective of their ability to pay, have access to the appropriate free ANC services for pregnant women.

There is evidence in the literature indicating that women who were less likely to practice family planning and those who did not seek care from a health professional were more likely not to use ANC services [27]. This is consistent with our finding that women who desired to get pregnant were more likely not to use ANC services.

Our findings may not represent the current situation of women in South Sudan and their need for access to reproductive care including ANC. As the result of the recent armed violence that broke out in the capital Juba on 15 December 2013, and which subsequently spread to several states in South Sudan, there has been further destruction of the health system. Since 15 December 2013, over 908,000 people have been displaced by violence, including 705,800 people within South Sudan and 202,500 into neighbouring countries [45]. The dead and the wounded are estimated to be in the tens of thousands [45,46]. As a result of this internal armed conflict the people of South Sudan have experienced severe health consequences exacerbated by population displacement, food insufficiency, and the collapse of the existing basic health services. For example the conflict has caused the collapse in the health system in Upper Nile state in the city of Malakal where, the hospitals that had survived two decades of civil war, have now been completely destroyed and looted. There is a similar situation in Jounglei, Upper Nile and Unity states that have all been severely affected by the conflict and created regional disparities within the country [47]. A drop in the number of women accessing reproductive health services as a result of this recent conflict was also reported [47]. Also lack of access to basic reproductive health services and the closure or destruction of health care facilities due to the violence has put the lives of women and their newborns at risk [48]. Further complicating the situation is the reduced access of these populations to life-saving health care from seasonal flooding. Nonetheless our findings remain important for future health care assessments once the conflict has ceased.

## Conclusion

Many underlying external environment, predisposing, need and enabling factors influence women’s non-use of maternal health services in South Sudan. This study found that the number of years of schooling influenced the women’s attitudes towards the use of ANC services. This is because the choice of the recommended number of ANC visits is influenced by education. Therefore the Ministry of Health and policy makers in South Sudan need to implement outreach programmes to raise the awareness about women’s education and to encourage South Sudanese women to pursue higher levels of education. It is also crucial for these programmes to target women from poor households and those from rural areas about the importance of ANC services, to increase their awareness as well to increase their use of these services. Strategies to increase access to health care services in rural areas should be a priority in South Sudan. Also implementing strategies that would reduce the financial burden associated with using maternal health services, such as, medical and transportation costs, would enable women from poor households to use maternal health services.
